# IR-Action Spectroscopy of the Astrochemically Relevant HCCS^+^ Cation

**DOI:** 10.1021/acsearthspacechem.5c00248

**Published:** 2026-01-06

**Authors:** Matteo Michielan, Kim Steenbakkers, Daniela Ascenzi, Jake A. Diprose, Miroslav Polášek, Sandra Brünken, Claire Romanzin, Brianna R. Heazlewood, Cristina Puzzarini, Vincent Richardson

**Affiliations:** † Dipartimento di Fisica, 19034 Universitá di Trento, I-38123 Trento, Italy; ‡ HFML-FELIX, Toernooiveld 7, 6525 ED Nijmegen, The Netherlands; § Institute for Molecules and Materials, 98810 Radboud University, Heyendaalseweg 135, 6525 AJ Nijmegen, The Netherlands; ∥ Department of Physics, 4591 University of Liverpool, L69 7ZE Liverpool, U.K.; ⊥ J. Heyrovský Institute of Physical Chemistry of the Czech Academy of Sciences, 182 00 Prague, Czechia; # 27048 Université Paris-Saclay, CNRS Institut de Chimie Physique, UMR8000, 91405 Orsay, France; ∇ Synchrotron SOLEIL, L’Orme de Merisiers, 91190 Saint Aubin, France; ° Department of Chemistry ”Giacomo Ciamician”, University of Bologna, Via P. Gobetti 85, Bologna, I-40129, Italy

**Keywords:** JWST, Sulfur Astrochemistry, Interstellar Medium, Thioketene, Dissociative Ionization

## Abstract

The thioketenyl cation (HCCS^+^) has been recently detected in the dark cloud TMC-1 by radioastronomical observations within the QUIJOTE survey. However, the infrared (IR) spectrum of this ion is yet to be reported in the literature. Spectroscopic reference data are essential for the search of HCCS^+^ using the James Webb Space Telescope, not only in molecular clouds and star-forming regions, but also in the ionospheres and upper atmospheres of exoplanets. In this work, we demonstrate a method for the selective generation of the HCCS^+^ ion in its triplet ground state (^3^Σ^–^) and use this method to obtain IR band positions for HCCS^+^. The IR-action spectrum of H_2_-tagged HCCS^+^ has been measured in a cryogenic 22-pole ion trap via IR photodissociation (IR-PD) spectroscopy with the FELIX light source in the wavenumber regions 450–1850 and 3000–3350 cm^–1^. Spectral information is complemented by theoretical calculations on the fragmentation mechanisms leading to the formation of HCCS^+^ from dissociative ionization of 2,5-dibromothiophene. The assignment of the experimental HCCS^+^ vibrational bands is aided by comparison with *ab initio* computed values from literature and from calculations at the UB3LYP/cc-pVQZ level of theory, for both the triplet (^3^Σ^–^) and singlet (^1^Σ^+^) states of HCCS^+^. The experimental HCCS^+^ spectra show an overall good agreement with the scaled theoretical values (to account for anharmonicity effects), facilitating assignment of the IR spectral features. These findings will enable new reactivity investigations and spectroscopic measurements to be conducted, and for HCCS^+^ to be included in astrochemical models and databases.

## Graphical abstract

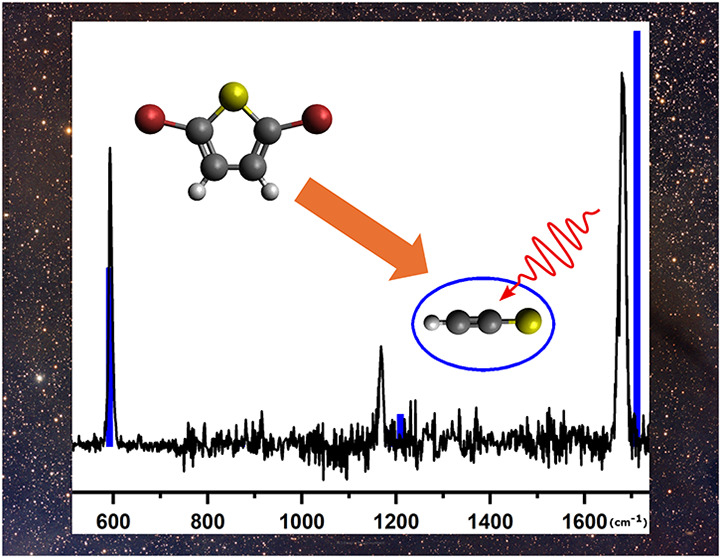

## Introduction

Sulfur is among the ten most abundant elements in astronomical environments, and interstellar sulfur chemistry is consequently a subject of growing interest. Recently, several inorganic and organic S-containing species have been detected in various astronomical sources spanning photodissociation regions, protoplanetary disks, diffuse and dense clouds, and exoplanetary atmospheres.^1–6^ These S-containing species display remarkable chemical diversity, ranging from hydrogenated compounds,^7–17^ unhydrogenated species,^12,18–20^ and saturated organics^21–23^ to molecules incorporating other heteroatoms such as N and O,^10,12,13,21,24–30^ and even organometallic species.^31^

While many molecular species are direct sulfur analogues of widely detected oxygen-containing molecules (e.g., CH_3_SH^23^ and CH_3_OH^32^ or CH_3_CHS^8^ and CH_3_CHO^33^), numerous oxygen-containing species lack identified sulfur-containing counterparts. Additionally, the observed abundances of S-containing species in dense molecular clouds and star-forming regions are significantly lower than those detected in warm, diffuse clouds and more primitive interstellar environments.^34–37^ Despite substantial modeling and observational efforts, the nature of this “missing” sulfur remains unresolved.^6,35^ The so-called “sulfur depletion problem” highlights the discrepancy between the high abundance of sulfur in the interstellar medium (ISM) and the relatively small fraction (∼8%) of observed S-containing molecules.^5,38–40^ This mismatch suggests that a significant portion of sulfur may be locked in undetected reservoirs (e.g., icy dust grains^41^) or in yet-to-be-observed chemical forms. The discrepancy is of both astronomical and astrobiological significance, as sulfur’s variable oxidation states and presence in a wide range of prebiotic molecules could play a crucial role in prebiotic astrochemistry.

A plethora of gaseous S-containing organic species have been detected in the carbon-rich cold dark cloud TMC-1, where conditions favor the formation of long carbon-chain radicals, cyanopolyynes, and cyclic hydrocarbons, including a number of sulfur-bearing carbon and hydrocarbon chains. In addition to the simple HCS/HSC isomers,^15^ organo-sulfur carbon and hydrocarbon chains such as HCCS, H_2_CCS, H_2_CCCS, C_4_S, C_5_S, HCCCCS, and HCSCCH,^12,13,42^ and even unsaturated cyclic molecules^9^ have been detected. In addition to neutral species, a number of S-containing cations have also been observed, including protonated C*_n_* S chains with *n* = 1–3.^11,14,16^ In particular, the thioketenyl cation (HCCS^+^) has recently been detected for the first time toward TMC-1 ^11,100^ through its rotational transitions occurring in the Q-band, observed with the Yebes 40 m radio telescope within the QUIJOTE spectral line survey. However, this assignment has been made through comparison between observed transitions and the previously calculated computational spectrum,^43,44^ since no laboratory spectroscopic measurements of this ion have so far been reported.

Previously,^45^ the formation of HCCS^+^ was thought to be driven by C–S bond-forming ion-neutral reactions such as S^+^ + C_2_H_2_, S + C_2_H_3_^+^, and S + C_2_H_4_^•+^. However, a more recent model^11^ concluded that the protonation of CCS is the major formation mechanism for HCCS^+^, with this pathway being underrepresented in the previous model due to an under-prediction of the density of CCS. Given the abundance of potential proton sources and the widespread nature of the CCS molecule—having not only been observed toward TMC-1 ^19^ but also toward several translucent clouds^46^ and in the circumstellar envelope of IRC + 10216 ^47^—the distribution of HCCS^+^ is expected to be similarly widespread.

Following the lines of our recent study on the H_2_CCS^•+^ radical cation,^48^ in this work, we report on the IR spectrum of HCCS^+^, which has been measured for the first time through IR photodissociation (IR-PD) spectroscopy. The selective formation of the HCCS^+^ ion at *m*/*z* 57 from 2,5-dibromothiophene fragmentation is confirmed through a combination of mass spectrometry and theoretical calculations involving both the fragmentation potential energy surface (PES) and the vibrational spectrum of HCCS^+^. The recorded spectrum is combined with saturation depletion measurements to quantify the purity of the mass-selected ion with regard to both isobaric contaminants and excited electronic states.

Addressing the high astrochemical interest in S-containing species, this work provides new spectroscopic data that could be used as a reference for future observations and missions, such as those involving the use of NIR and MIR spectrometers of the James Webb Space Telescope (JWST) in detecting sulfur-bearing species in the ionospheres and upper atmospheres of exoplanets.^49,50^ Furthermore, this work enables the identification of the dissociative ionization of 2,5-dibromothiophene as a selective method for the generation of HCCS^+^ for future reactivity and/or spectroscopic studies.

## Materials and Methods

### Experimental Methodology

Experiments were conducted using the FELion cryogenic ion trap apparatus at the Free Electron Lasers for Infrared eXperiments (FELIX) Laboratory,^51^ which has been described in detail previously.^52^ HCCS^+^ ions were generated via the dissociative ionization of 2,5-dibromothiophene (c-SCBrCHCHCBr, Sigma-Aldrich, ≥95%), which was evaporated into the setup from a liquid sample. Ionization was performed using both a direct electron ionization (EI) source and a storage ion source (SIS). For the EI source, ion source pressures were maintained at approximately 3 × 10^–5^ mbar with electron energies of ∼36 eV, while, in the case of the SIS, typical ion source pressures were on the order of 5 × 10^–5^ mbar with electron energies in the 30–40 eV range. A mass spectrum showing the generation of the *m*/*z* 57 fragment corresponding to [C_2_HS]^+^, as well as other products of the dissociative ionization process, using the EI source, is reported in Figure 1. Mass spectra obtained with the SIS source and a comparison of the performances of the two sources are given in the Supporting Information.

**Figure 1:**
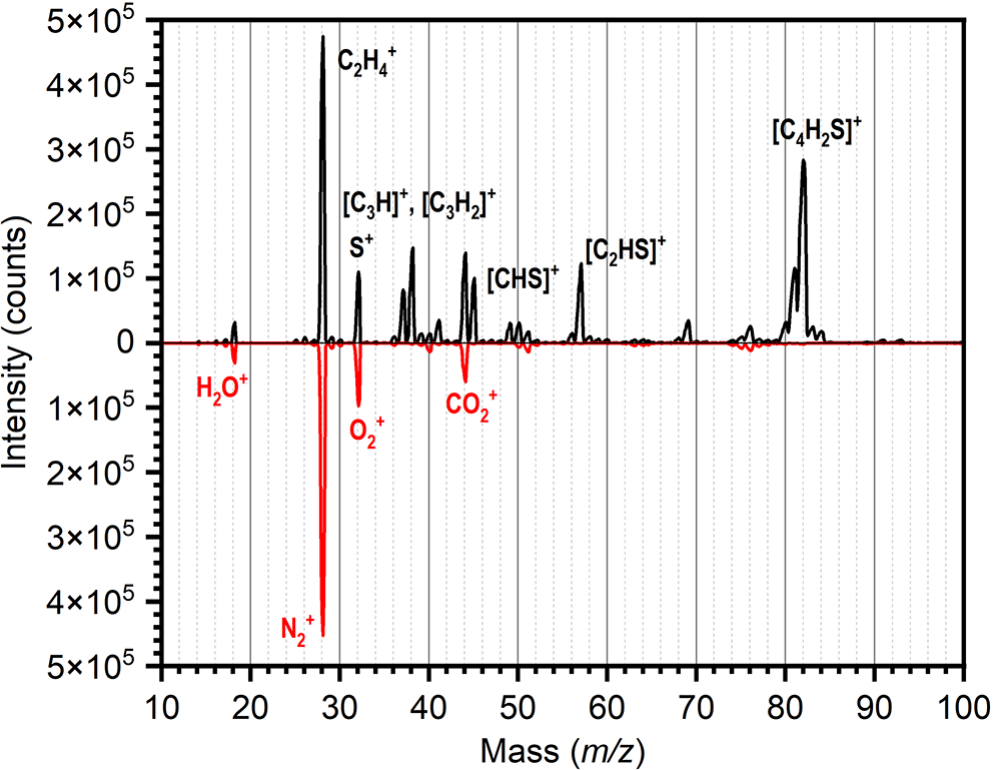
Mass spectrum of the ions formed from 2,5-dibromothiophene using the ∼36 eV EI source (black), compared to the background spectrum for this source (red, reflected below the axis) recorded prior to admission of 2,5-dibromothiophene. Labels in red are given for the major background ions, while labels in black are given for both the major peaks arising from the dissociative ionization of 2,5-dibromothiophene and notable species that are isobaric with respect to the major background peaks.

Following their generation, *m*/*z* 57 ions were mass-selected and extracted using a quadrupole mass filter. The selected ions were then guided into a cryogenic 22-pole trap stabilized at a nominal temperature of ∼12 K. Ions were cooled to the trap temperature through collisions with a 98.5:1.5 mixture of He:H_2_, introduced through a pulsed piezo valve at a number density of ∼10^14^–10^15^ cm^–3^. Each pulse was triggered 15 ms before the ions entered the trap and lasted for the duration of the ion pulse, enabling a significant fraction (∼15%) of the primary ions to form weakly bound complexes with H_2_. Comparison between the mass spectra of the trapped, mass-selected *m*/*z* 57 ion fragments before and after H_2_-tagging is shown in Figure 2. Although a small contribution from multiply tagged ions was observed at successive 57 + 2*n* masses (where *n* is the number of attached H_2_), the number density and composition of the gas pulse were adjusted to maximize the relative intensity of the singly tagged (*m*/*z* 59) signal, with the small contribution from multiply tagged ions not expected to impact the recorded spectra.

**Figure 2:**
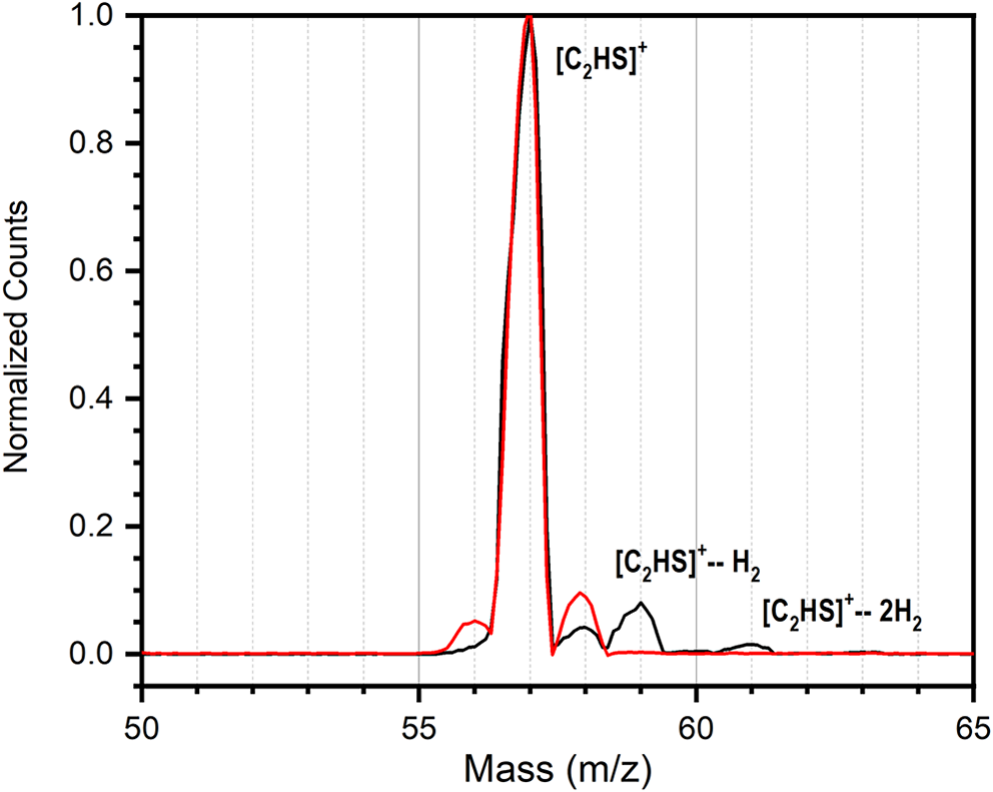
Normalized mass spectra of mass-selected *m*/*z* 57 ions generated via the dissociative ionization of 2,5-dibromothiophene using the EI source, both before (red) and following H_2_-tagging of these same ions (black). The decrease in *m*/*z* 56 and 58 signals in the tagged spectrum is an effect of the normalization.

IR-PD spectra are measured as a depletion of the singly tagged (*m*/*z* 59) ion signal as a function of the laser frequency, with the absorption of resonant photons leading to the dissociation of the weakly bound complex ions. IR radiation was provided by the FEL2 free electron laser of the FELIX Laboratory, which was operated at 10 Hz with typical macropulse energies of 3–8 mJ in the trap region. The recording of spectra in this way also prevents any impact from neighboring masses (for example, the small peak observed at *m*/*z* 58 in Figure 2) on the recorded spectra. The spectra presented here are given in terms of the measured depletion intensity on a per-photon basis, which allows for a normalization of the differences in laser power at different wavenumbers. Normalization of the signal intensity *I* is given in the following equation

1 $$I=-\frac{\ln\left(S/B\right)\cdot h\nu }{E\cdot N}$$

where *S* is the observed ion counts, *B* is the baseline counts, *h*ν is the photon energy, *E* is the laser energy of a single pulse (in mJ), and *N* is the number of pulses. Depletion saturation measurements were performed at different wavelengths and using both the EI and SIS sources, in order to quantify the purity of the mass-selected ion with regard to both isobaric contaminants and excited electronic states. The method has previously been described in detail^52,53^ and involves irradiation of the tagged ions with a sequence of laser pulses resonant with a species-specific vibrational band, leading to the complete dissociation of all H_2_-tagged complexes of a given species. This process allows for differentiation between isobaric contaminants, isomeric species, or different electronic states of the same isomer. The fractional abundance of the relevant species, *A*, is then determined via the fitting of the tagged mass ion signal as a function of the deposited energy using the following equation

2 $$D=1-\frac{S}{B}=A\cdot \left(1-e^{-k\cdot E\cdot N}\right)$$

where *D* is the depletion percentage and *k* is the efficiency of the IR-PD process for the band being probed. For all depletion values presented in this work, the calculated error is the sum of the fitting error and a systematic 5% uncertainty arising from a combination of detection noise and insufficient overlap between the irradiation and trapping regions.

### Theoretical Methodology

Electronic structure calculations were performed to identify the fragmentation mechanisms leading to the formation of [C_2_HS]^+^ from 2,5-dibromothiophene. Calculations of the structures and energies of the reagent, products, intermediates, and transition states have been carried out using the UB3LYP/cc-pVQZ level of theory. Harmonic frequency calculations were used to characterize the stationary points and identify local energy minima (where all frequencies are real) and first-order saddle points (one imaginary frequency). Frequencies were then used to calculate zero-point vibration energy (ZPVE) corrections. Improved energies are obtained using the G4 method,^54^ with several test calculations of ionization energies performed and compared with experimental data to assess the performance of the UB3LYP/cc-pVQZ and G4 methods for several relevant sulfur-containing ions.

Geometry optimization and subsequent harmonic and anharmonic vibrational frequency calculations for the triplet (^3^Σ^–^) and singlet (^1^Σ^+^) states of HCCS^+^ (*C*_∞*v*_) have been performed at the UB3LYP level of theory, using both cc-pVTZ and cc-pVQZ^55,56^ basis sets, with further details on the excited state calculations given in the Supporting Information. We have employed a scaling factor of 0.951 for the UB3LYP harmonic vibrational frequency calculations, which has been determined by comparing the experimental spectrum with the calculations performed in this study.

In addition, the geometry of the HCCS^+^-H_2_ complex was optimized at the UB3LYP level using the cc-pVTZ, cc-pVQZ, aug-cc-pVTZ, and aug-cc-pVQZ basis sets,^55–57^ which include diffuse functions to model long-range dispersive interactions. Counterpoise corrections were performed to account for basis set superposition errors (BSSEs).^58,59^ In order to account for dispersion forces, further calculations were performed using the empirical D3 version of Grimme’s dispersion with Becke-Johnson damping^60^ in connection with the UB3LYP functional.

Detailed results for the calculation of ionization enthalpies, details on the scaling factor determination, and predicted spectra obtained both at different levels of theory and for the tagged complex are provided in the Supporting Information. All calculations mentioned in this section have been performed using the Gaussian 16 suite of programs.^61^

## Results

### IR-PD Spectroscopy of HCCS^+^

The experimental IR-PD spectrum of the H_2_-tagged *m*/*z* 57 ion generated using the SIS source in the 450–1850 and 3000–3350 cm^–1^ ranges is shown in Figure 3 alongside the harmonic and anharmonic UB3LYP frequencies calculated as part of this work. Comparison of the spectral features of the SIS and EI sources is given in the Supporting Information, from which we conclude that the same ions are generated using the two sources. Only data from the SIS source are presented here due to the better signal-to-noise ratio. The line positions, intensities, and widths of the various experimental and computed features—obtained by fitting the experimental spectrum with Gaussian functions—are detailed in Table 1. The relative intensities of the experimental features have been normalized with respect to the harmonic UB3LYP/cc-pVQZ calculations using the C–H stretching band of the ground (triplet) state. Comparison between the experimental spectrum and the calculated features (established using a range of different levels of theory, including harmonic calculations at the CCSD­(T)/cc-pwCVQZ level from ref 44), is given in the Supporting Information.

**Figure 3:**
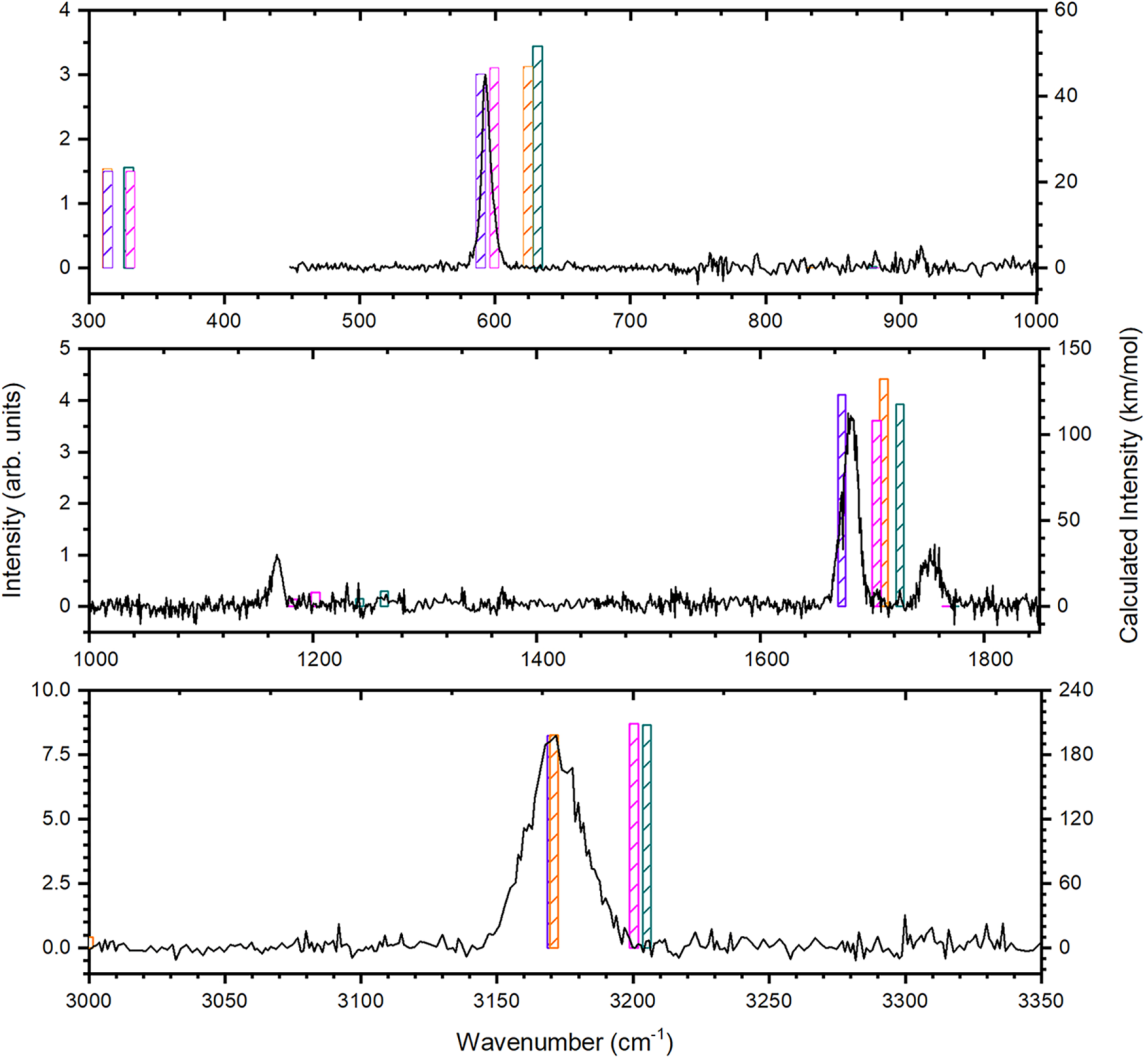
IR-PD vibrational spectra of the H_2_-tagged *m*/*z* 57 fragment from dissociative ionization of 2,5-dibromothiophene: experimental results are given in black while spectra of the bare ion computed at the UB3LYP/cc-pVQZ level are shown in purple (^3^Σ^–^, scaled harmonic), pink (^3^Σ^–^, anharmonic), orange (^1^Σ^+^, scaled harmonic), and dark cyan (^1^Σ^+^, anharmonic) lines.

**Table 1:** Experimental IR Band Positions,^a^ Intensities (in Relative Units), Widths, and Depletion Percentages are Compared with Calculated Values of the Bare Ion for the Triplet (^3^Σ^–^) and Singlet (^1^Σ^+^) HCCS^+^ Ions^b^.

mode	assignment	symmetry	IR-PD Pos. (cm^–1^)	IR-PD Int. (rel. u.)	line width (cm^–1^)	depletion %	Calc. Pos.^c^ (cm^–1^)	Calc. Int.^c^ (km·mol^–1^)	Calc. Pos.^d^ (cm^–1^)	Calc. Int.^d^ (km/mol)
HCCS^+^ (^3^Σ^–^)										
										
C–C–S bend	ν_1_	π	-	-	-	-	313	22.6	330	23.2
H–C–C bend	ν_2_	π	594(3)	24(1)	7(2)	93(8)	589	45.2	599	46.6
C–S stretch	ν_3_	σ	-	-	-	-	836	0.02	880	0.1
H–C–C bend, overtone	2ν_2_, l = 0	π	1168(5)	9(1)	9(6)	-	1179	-	1182	4.2
H–C–C bend, overtone	2ν_2_, l = ±2	π							1203	8.4
C–C stretch	ν_4_	σ	1681(4)	62(2)	14(3)	89(6)	1675	123.2	1704	108.3
C–S stretch, overtone	2ν_3_	σ	1753(9)	18(2)	16(15)	-	1672	-	1767	0.2
C–H stretch	ν_5_	σ	3171(4)	197(4)	21(4)	89(6)	3170	197.7	3200	208.8
HCCS^+^ (^1^Σ^+^)										
										
C–C–S bend	ν_1_	π	-	-	-	-	313	23.2	329	23.5
H–C–C bend	ν_2_	π	-	-	-	-	624	46.9	631	51.6
C–S stretch	ν_3_	σ	-	-	-	-	833	0.2	878	0.3
H–C–C bend, overtone	2ν_2_, l = 0	π	-	-	-	-	1247	-	1242	4.4
H–C–C bend, overtone	2ν_2_, l = ±2	π							1264	8.9
C–S stretch, overtone	2ν_3_	σ	-	-	-	-	1667	-	1774	0.2
C–C stretch	ν_4_	σ	(1753(9))	(18(2))	(16(15))	-	1702	132.5	1725	117.8
C–H stretch	ν_5_	σ	-	-	-	-	3171	198.1	3205	207.3

a Experimental values have been determined by fitting the spectrum with multiple Gaussian functions, with the intensity obtained as the area of a given peak. Uncertainties are provided in parentheses in the last digit.

b Overtone positions obtained from the harmonic calculations are multiples of the fundamental position. The calculated intensities for degenerate modes are given as the sum of the degenerate modes to ease comparison with experimental intensities.

c This work, UB3LYP/cc-pVQZ (harmonic). For comparison with experimental results, a vibrational scaling factor of 0.951 has been applied to the calculated frequencies.

d This work, UB3LYP/cc-pVQZ (anharmonic).

Overall, we observe a high level of agreement between the experimental band positions and those predicted by both harmonic and anharmonic calculations for the ground (triplet) state of HCCS^+^. The lowest wavenumber features computed for both the singlet and triplet states are outside the range studied in this work, but the intense low-wavenumber band observed at 594 cm^–1^ matches well with the computed position of the triplet H–C–C bend of 589­(scaled harm.)/599­(anharm.) cm^–1^. Similarly, the primary feature observed in the intermediate-wavenumber region at 1681 cm^–1^ matches well with a predicted triplet band, namely the C–C stretch at 1675­(scaled harm.)/1704­(anharm.) cm^–1^. Finally, in the high-wavenumber region, the observed band at 3171 cm^–1^ is in excellent agreement with the predicted position of the triplet C–H stretch at 3170­(scaled harm.)/3200­(anharm.) cm^–1^. While we do not observe any intense features in the region of the 836­(scaled harm.)/880­(anharm.) cm^–1^ C–S stretching band predicted by the calculations, this is trivially explained by the predicted low intensity of 0.02­(harm.)/0.06­(anharm.) km·mol^–1^.

In addition to the features predicted by harmonic calculations, we also observe a pair of additional features in the intermediate-wavenumber region. The first of these is at 1168 cm^–1^, with a second feature at 1753 cm^–1^. The latter is potentially a match for the predicted C–C stretching band of the singlet state at 1756 cm^–1^, but no harmonic peak is predicted in the region of the former for either the singlet or triplet state. However, both features are in reasonable agreement with the first overtones of the H–C–C bend and C–S stretch, respectively, which would be anticipated to occur at 1179 and 1672 cm^–1^ on the basis of the harmonic calculations. This is supported by the anharmonic calculations, where overtones are predicted at 1182, 1203, and 1767 cm^–1^, with the splitting of the first overtone in the anharmonic calculations being the result of *l*-doubling. Further consideration of these intermediate-region features is given in the Discussion section.

The relative intensities of the bands for which clear assignments have been made are broadly in agreement with those predicted by calculations, though we note that only the anharmonic calculations are able to provide intensities for the overtone features. The experimental, 0.39(3), and calculated (0.36 for harmonic and 0.43 for anharmonic) relative intensities of the 594 cm^–1^ band with respect to the 1681 cm^–1^ band are in good agreement, with both features being comparatively under-observed experimentally with respect to the 3171 cm^–1^ band. The calculated summed relative intensity of the nondegenerate overtones around 1170 cm^–1^ with respect to the 594 cm^–1^ fundamental of 0.27 is in fair agreement with the value of 0.38(6) observed experimentally. However, the calculated relative intensity of the overtone feature at 1767 cm^–1^ with respect to the fundamental is approximately 2 orders of magnitude lower than that observed experimentally, though we note that the predicted intensity of this feature is greater than that of the fundamental, consistent with what is observed experimentally. Further consideration of this point is also given in the Discussion section.

To further investigate the spectral characteristics and to quantify the fractional abundance of the triplet and singlet states, depletion saturation measurements were performed at 590 (using both EI and SIS sources), 1692, and 3171 cm^–1^. The full details of these measurements are given in the Supporting Information, with the results summarized in the Depletion column of Table 1. The observed depletion fits are consistent with the presence of a single species comprising approximately 90(7)% of the observed *m*/*z* 57 fragment signal. This supports the assignment of the predominant observed bands to a single species, namely the HCCS^+^ ion in the triplet (^3^Σ^–^) ground state.

### Fragmentation Potential Energy Surface of 2,5-Dibromothiophene

The PES calculations presented here focus on the fragmentation channels from the 2,5-dibromothiophene radical cation (c-SCBrCHCHCBr^•+^) to give HCCS^+^, rather than exploring the full dissociative PES. The PES is shown graphically in Figure 4, while the structures of the relevant identified intermediate structures and transition states are provided in the accompanying data repository (see the Data Availability Statement). In the following description, energies are given in parentheses, in kJ·mol^–1^, relative to the energy of the dibromothiophene cation. Previous investigations on the structures, energies, and harmonic vibrational frequencies^43,44^ found the geometry of the HCCS^+^ ion to be linear, with a ^3^Σ^–^ ground electronic state and a ^1^Σ^+^ excited state.

**Figure 4:**
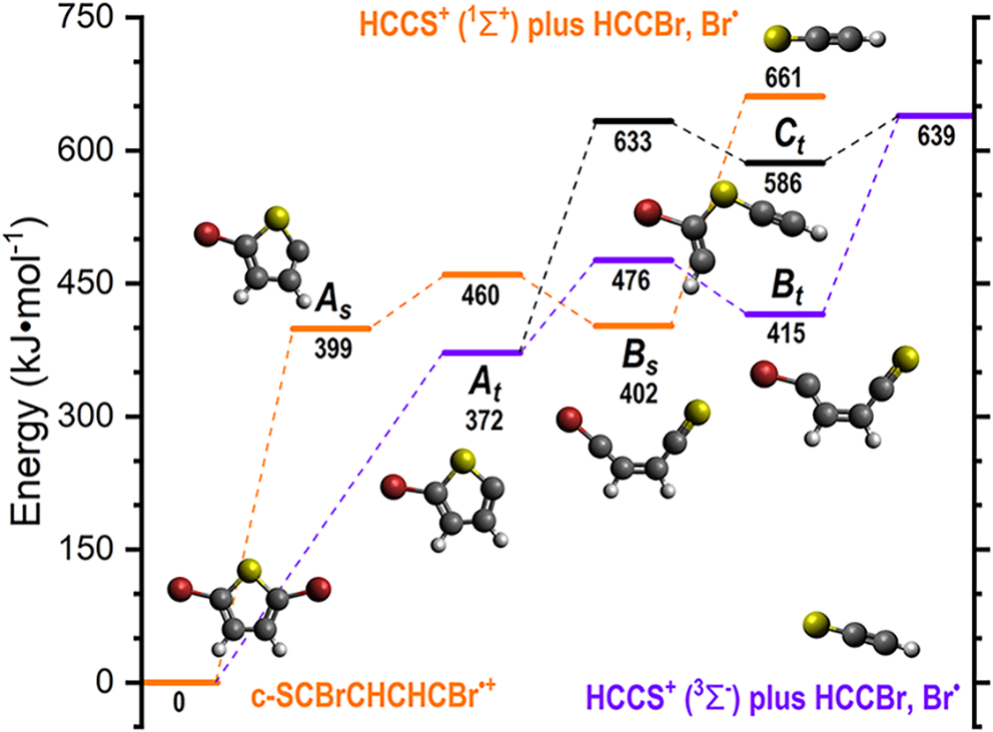
Calculated fragmentation scheme of the precursor 2,5-dibromothiophene radical cation to form the HCCS^+^ ion in both the triplet and singlet forms.

The fragmentation of c-SCBrCHCHCBr^•+^ to give HCCS^+^ involves an initial cleavage of one of the C–Br bonds, leading to the ejection of Br^•^ to give c-SCCHCHCBr^+^. This ion can exist as either a *C*_*s*_-symmetric triplet (372), hereafter referred to as *A*_*t*_, or a *C*_1_-symmetric singlet (399), hereafter referred to as *A*_*s*_. The relaxed two-dimensional PES scan (corresponding to the elongation of one of the two equivalent C–Br bonds) indicates that the loss of Br is continuously endothermic, with the lower energy triplet state expected to form preferentially.

From *A*_*t*_, the lowest energy pathway leading to HCCS^+^ involves an initial C–S bond cleavage via a transition state located 476 kJ·mol^–1^ above c-SCBrCHCHCBr^•+^, to give SCCHCHCBr^+^ as a *C*_*s*_-symmetric triplet (415), hereafter referred to as *B*_*t*_. *B*_*t*_ can then eject bromoethyne (HCCBr, a *C*_∞*v*_-symmetric singlet) via C–C cleavage to give HCCS^+^ as a *C*_∞*v*_-symmetric triplet (639). These same products can also be reached from *A*_*t*_ through an initial C–C cleavage via a transition state located 633 kJ·mol^–1^ above c-SCBrCHCHCBr^•+^, to give HCCSCBrCH^+^ as a *C*_*s*_-symmetric triplet (586), hereafter referred to as *C*_*t*_. Like *B*_*t*_, *C*_*t*_ can also proceed to give HCCS^+^ as a *C*_∞*v*_-symmetric triplet in combination with HCCBr as a *C*_∞*v*_-symmetric singlet without a barrier, this time via C–S bond cleavage. However, given the higher energies of the transition state and the *C*_*t*_ intermediate, the contribution from this channel is expected to be minimal.

The higher energy singlet state of HCCS^+^ can form exclusively from *A*_*s*_ through an initial C–C cleavage via *A*_*s*_-*B*_*s*_ (460) to give SCCHCHCBr^+^ as a *C*_*s*_-symmetric singlet (402), hereafter referred to as *B*_*s*_. *B*_*s*_ can then eject bromoethyne (HCCBr, again as a *C*_∞*v*_-symmetric singlet) via C–C cleavage to give HCCS^+^ as a *C*_∞*v*_-symmetric singlet (661) without a barrier. Attempts to identify a singlet equivalent to *C*_*t*_ proved unsuccessful.

## Discussion

The main features of the measured IR-PD spectra show a high level of agreement with the computed scaled harmonic and anharmonic vibrational bands of the bare ion obtained at the UB3LYP/cc-pVQZ level of theory for the triplet ground state of HCCS^+^. The experimental H–C–C bend, C–C stretch, and C–H stretches are all less than 5 cm^–1^ from their predicted positions for the harmonic calculations, with only slightly larger deviations observed for the anharmonic calculations. In all cases, the deviations are well within the experimental line widths (see Table 1). In terms of intensities, while the C–H stretch is comparatively more intense experimentally compared to calculations, the relative intensities of the H–C–C bend and C–C stretch are in excellent agreement with calculations, with the failure to experimentally observe the C–S stretch explained by the very low predicted intensity.

When considering the overtone features predicted by the anharmonic calculations of the bare ion, there is good agreement between the observed features at 1168 and 1753 cm^–1^ with the predicted overtone features at 1182, 1203, and 1767 cm^–1^ (see Table 1 for assignments). However, while the anharmonic calculations predict the first overtone to be nondegenerate—giving rise to predicted features at both 1182 and 1203 cm^–1^—we observe only a single experimental feature in this region. This splitting is the result of *l*-doubling, with the 1182 cm^–1^ feature corresponding to *l* = 0 and the 1203 cm^–1^ feature corresponding to *l* =±2. However, we are unable to determine whether the observation of a single experimental peak is due to the splitting between the two peaks being much smaller than predicted (so they appear as a single peak), or because the intensity of one band is lower than predicted and therefore it is not observed. A further possibility is that the H_2_ tag could break the linear symmetry and so remove any *l*-doubling effect, please see the Supporting Information for more details. Nevertheless, the agreement between the experimental results and anharmonic calculations for this feature is sufficient to confidently assign it to an overtone. From the depletion saturation measurements performed on the 594 and 1681 cm^–1^ bands, we therefore conclude that the triplet ground state accounts for ∼90% of the observed *m*/*z* 57 signal.

A minor contribution from the singlet excited state cannot be entirely ruled out. As this is expected to account for ≤10% of the observed *m*/*z* 57 signal, only the most intense bands are likely to be detected experimentally. In the following paragraph, only calculated values from the anharmonic calculations are provided for clarity; please see Table 1 for the corresponding harmonic values. From calculations, the most intense band is the C–H stretch at 3205 cm^–1^ with an intensity of 207.3 km·mol^–1^. However, this cannot be clearly identified due to its overlap with the predicted position of the ground state C–H stretch at 3171 cm^–1^, as both fall within the experimental line width (21 cm^–1^) of the observed band. The next most intense predicted band is the C–C stretch at 1725 cm^–1^ with an intensity 117.8 km·mol^–1^, which is in reasonable agreement with the observed band at 1753 cm^–1^, for which the ground state overtone band, predicted by anharmonic calculations, is expected to be weak (0.2 km·mol^–1^). However, we note that as the absolute depletion intensity for this feature is more than 10%, some contribution from the overtone is present. Finally, the H–C–C bend at 631 cm^–1^ with a predicted intensity of 51.6 km·mol^–1^ is at odds with the absence of an experimental feature in this region. Given the comparatively high predicted intensity of this band, we therefore conclude that the overall contribution from the excited state is minor, in agreement with the depletion results.

Although no significant deviations between the experimental and calculated line positions are observed, indicating a limited influence of the H_2_ tag on the observed spectrum, we have performed further calculations (at the B3LYP/cc-pVQZ level) on the tagged complexes, with detailed results provided in the Supporting Information. In brief, we note that the calculations of the tagged complex show a broad level of agreement with experiment, but the breaking of the linear symmetry leads to a loss of degeneracy for the H–C–C bending mode that results in a splitting of the feature that is not observed experimentally, but which we cannot disregard being sufficiently small to be unresolvable. However, the anharmonic tagged calculations do accurately reproduce both the positions and relative intensities of the two observed overtones.

Results from the calculated PES further support the assignment of approximately 90% of the *m*/*z* 57 ions to the triplet ground state of HCCS^+^. In fact, both the ground and the excited state formation pathways are predicted to be continuously endothermic, but the ground state route lies 22 kJ·mol^–1^ lower in energy. These findings indicate that even greater suppression of the excited state could be achieved by exercising tighter control over the ionization process, for example, by using photoionization via a tunable VUV source.

Finally, we note that while different levels of theory are broadly consistent in predicting line positions, they yield significantly different relative intensities. This is outlined in more detail in the Supporting Information, where the experimental spectrum is compared to calculations at various levels of theory. The best agreement with the experimental data is found for calculations performed at the UB3LYP/cc-pVQZ level; the previously reported CCSD­(T) results^44^ fail to accurately reproduce the observed intensities. We are currently unable to explain this discrepancy between experiment and the CCSD­(T) predictions, and resolving this issue will require further investigation beyond the scope of this work.

## Conclusions

This study marks the first experimental spectrum of the thioketenyl cation (HCCS^+^), with the IR action spectrum of the H_2_-tagged ion having been recorded using a cryogenic 22-pole ion trap and IR photodissociation (IR-PD) spectroscopy, employing the FELIX light source in the 450–1850 cm^–1^ and 3000–3350 cm^–1^ spectral regions. Spectral features—including band positions, intensities, and widths—are interpreted in conjunction with saturation depletion measurements and supported by theoretical calculations on the fragmentation mechanisms leading to HCCS^+^ formation via dissociative ionization of 2,5-dibromothiophene.

Consistent with previous theoretical work, the HCCS^+^ ion is found to have a linear geometry, with a ^3^Σ^–^ground electronic state and a ^1^Σ^+^ excited state located approximately 22 kJ·mol^–1^ above the ground state. Vibrational band assignments are guided by comparisons with *ab initio* values reported in the literature and with new calculations performed at the UB3LYP/cc-pVQZ level for both the triplet (^3^Σ^–^) and singlet (^1^Σ^+^) states of HCCS^+^. Good agreement is found between the experimental data and both scaled harmonic frequencies (to account for anharmonicity) and anharmonic calculations. The main outcomes of this study can be summarized as follows:

- The triplet state of HCCS^+^ accounts for approximately 90% of the observed *m*/*z* 57 fragment signal resulting from dissociative electron ionization of 2,5-dibromothiophene, regardless of the ion source used.
- Electron ionization coupled with cryogenic cooling is an effective method for selectively generating the electronic ground state (^3^Σ^–^) of HCCS^+^. This experimental approach can now be employed to selectively generate HCCS^+^ for both high-resolution spectroscopy and detailed astrochemical reactivity studies. The latter are essential for the effective modeling of the role played by this species in the overall chemical evolution of astrochemical environments.
- Reliable IR band positions for the thioketenyl cation are reported. This, combined with the formation method characterized here, will enable future high-resolution spectroscopy measurements of this ion, for example, using the recently developed leak-out spectroscopy method.^62–65^ Although this ion has recently been detected in the TMC-1 dark cloud via radioastronomical observations, high-resolution experimental spectroscopic data, especially in the IR region, are essential for its identification using the James Webb Space Telescope, not only in molecular clouds and star-forming regions, but also in the ionospheres and upper atmospheres of exoplanets.

## Supplementary Material



## Notes

Comparison of the two ionization sources used in this work, full results of the depletion measurements, scaling factor determination, comparison of predicted spectra at different levels of theory and with the inclusion of the tag, details of the excited singlet state calculations, and geometries for the minima and transition states identified in the PES. The experimental data used to support the findings of the paper are openly available in https://doi.org/10.17638/datacat.liverpool.ac.uk/3050 (PDF)
