# Supplementary material for: IR-Action Spectroscopy of the Astrochemically Relevant HCCS+ Cation
Source: ACS Earth Space Chem. 2026 Jan 6;10(1):148–56. doi: 10.1021/acsearthspacechem.5c00248 (PMC12814772; doi:10.1021/acsearthspacechem.5c00248)
Supplement: Supplementary file 1 [file sp5c00248_si_001.pdf]

Supporting Information for Publication: IR-Action Spectroscopy of the astrochemically relevant $HCCS^+$ cation

Matteo Michielan,[†] Kim Steenbakkers,[‡] Daniela Ascenzi,[†] Jake Diprose,[¶] Miroslav Polášek,[§] Sandra Brünken,[‡] Claire Romanzin,[‖] Brianna R. Heazlewood,[¶] Cristina Puzzarini,[⊥] and Vincent Richardson[*,¶]

†*Dipartimento di Fisica, Universitá di Trento, I-38123 Trento, Italy*

‡*HFML-FELIX, Toernooiveld 7, 6525ED Nijmegen, the Netherlands/Institute for Molecules and Materials, Radboud University, Heyendaalseweg 135, 6525 AJ Nijmegen, the Netherlands*

¶*Department of Physics, University of Liverpool, Liverpool, UK*

§*J. Heyrovský Institute of Physical Chemistry of the Czech Academy of Sciences, Prague, Czechia*

‖*Université Paris-Saclay, CNRS, Institut de Chimie Physique, Orsay, France/Synchrotron SOLEIL, L'Orme de Merisiers, Saint Aubin, France*

⊥*Department of Chemistry "Giacomo Ciamician", University of Bologna, Via P. Gobetti 85, Bologna, I-40129, Italy*

E-mail: vincent.richardson@liverpool.ac.uk

Comparison of EI and SIS sources

The data presented in this work were collected over two beamtimes in May and October 2023. In the former, a direct electron ionization (EI) source was employed, while a storage ion source (SIS) was employed during the second beamtime. In order to compare the fragmentation patterns observed with the two sources, and to verify that the spectra obtained can be treated without specification, we present mass and vibrational spectra obtained with both sources.

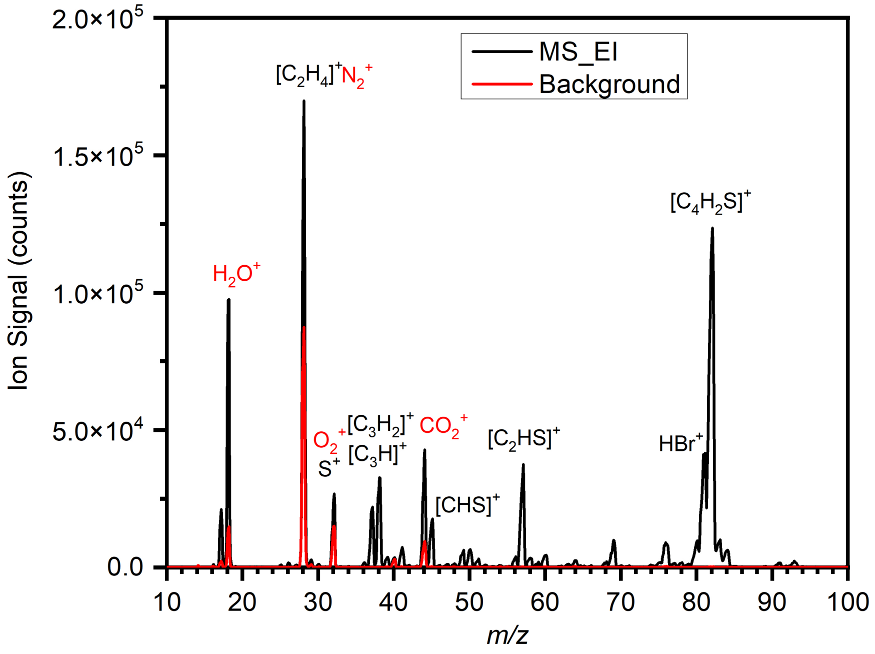

Figure S1: Mass spectrum of ions generated by the EI source both before (red) and after (black) admission of 2,5-dibromothiophene.

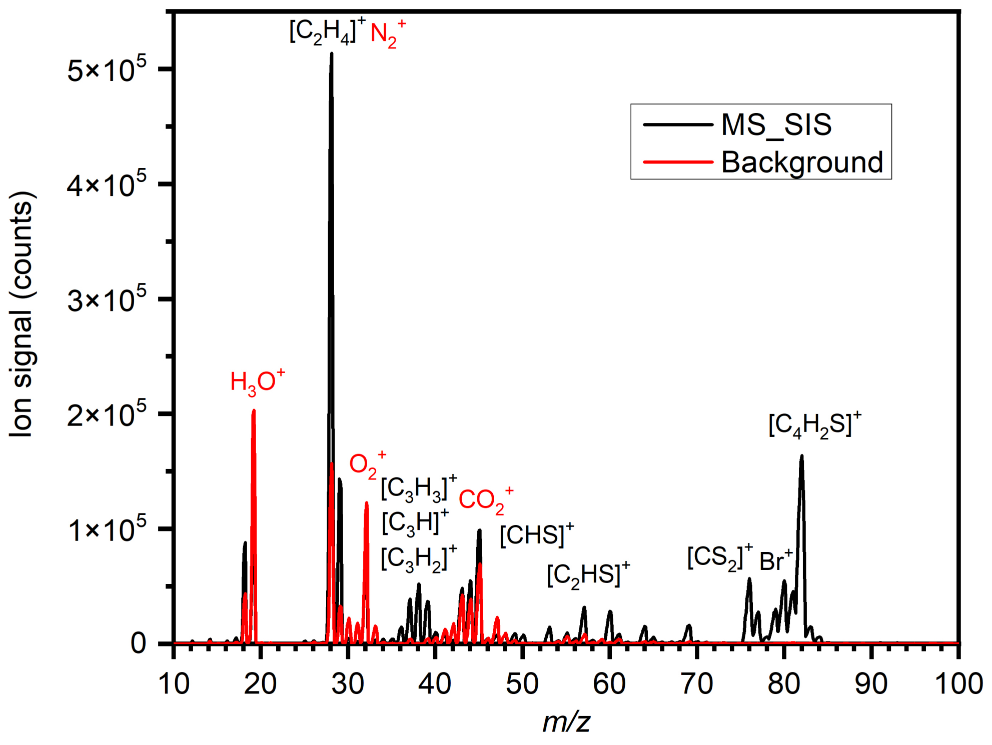

Figure S2: Mass spectrum of ions generated by the SIS source both before (red) and after (black) admission of 2,5-dibromothiophene.

A representative mass spectrum for the ions formed using the EI source is shown in Figure S1, with an equivalent spectrum for the SIS source shown in Figure S2. In both cases, the background mass spectrum is shown in red, with the mass spectrum following admission of 2,5-dibromothiophene in black. As a general characteristic of the source, the mass spectrum collected with the SIS exhibits a higher proportion of products corresponding to the enhanced fragmentation of the 2,5-dibromothiophene ring as well as a wider variety of background species. This is expected, as the longer storage time allows for the initially-formed ions to undergo reactions with neutral 2,5-dibromothiophene, as evidenced by the m/z 76 peak corresponding to the formation of carbon disulfide radical cation $[CS_2]^{+\bullet}$, which is barely present in the case of the EI source. Furthermore, the longer trapping times also lead to an enhancement of protonated species, as evidenced by the observation of $H_2O^{\bullet+}$ using the EI source and H_3O^+ using the SIS source.

Nonetheless, in both cases we observe a quite intense m/z 57 fragment corresponding to

the formation of $[C_2HS]^+$, although the relative intensity of this ion is reduced when working with the SIS. We further note that the use of the dibrominated thiophene effectively enhances the relative intensity of the *m/z* 57 fragment channel, with the *m/z* 58 fragment channel being the dominant fragment for the dissociative ionization of non-brominated thiophene.[1] To verify that the species formed does not differ between the two sources, a comparison of the spectra obtained using the two sources in the 500-1000 cm^{-1} region is given in Figure S3, with May EI source data shown in black and the October SIS source data in red. While we observe a consistent difference in the noise levels for the two spectra, the high level of agreement allows us to conclude that the same chemical species is generated in both cases. This finds further confirmation after noting the purity obtained using depletion saturation measurements of the ~590 cm^{-1} feature which were performed using both sources, results of which are shown in the Section relating to depletion saturation measurements.

The minor change in relative intensities observed in Figure S3 is attributed to the significant laser power instabilities affecting the measurements during the May beamtime. However, we note that such a noise introduction is significantly reduced when normalizing the spectrum with respect to the laser power.

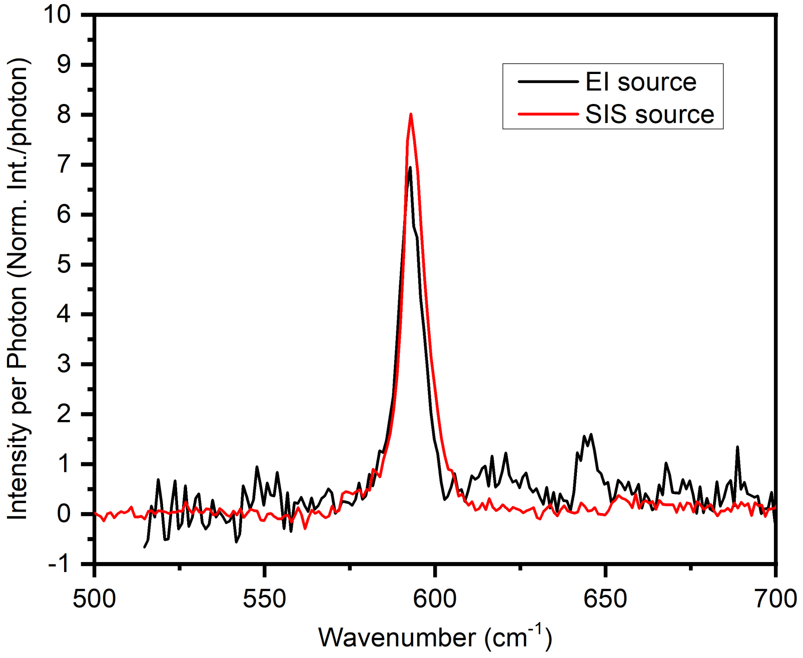

Figure S3: Intensity per photon IRPD spectra of $[HCCS]^+$ fragments from data collections from May (black) and October (red) beamtimes.

Depletion saturation measurements

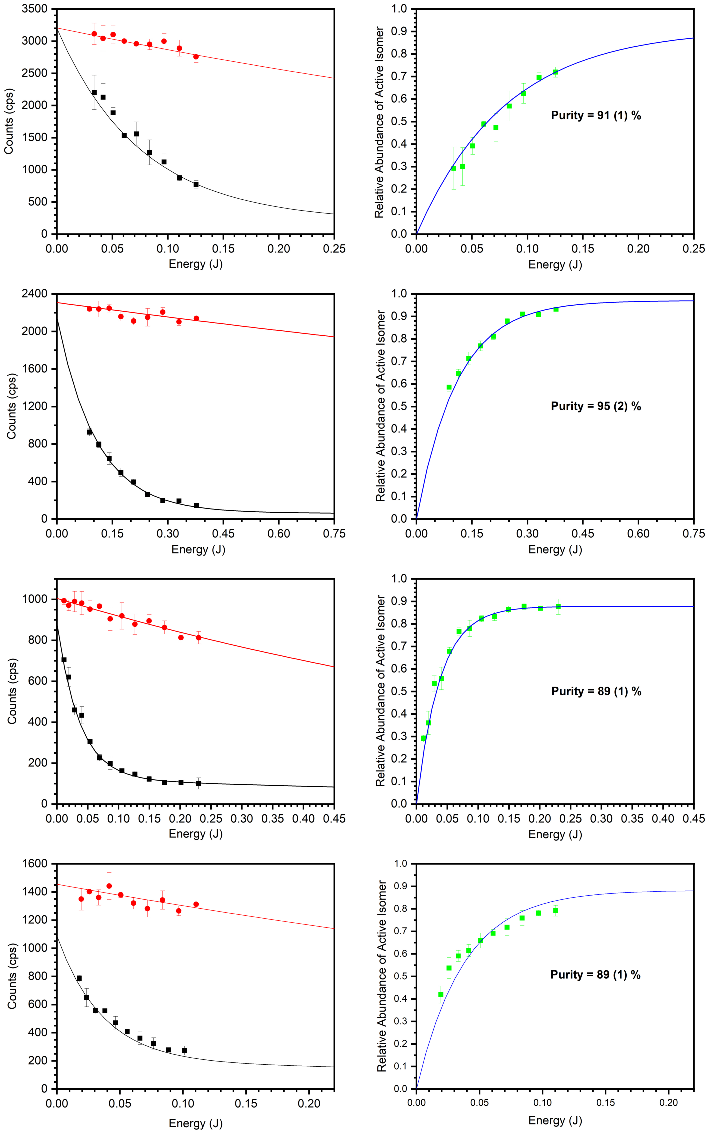

Figure S4: Depletion analysis for both the low wavenumber region recorded using the EI source and the low, intermediate and high wavenumber regions recorded using the SIS source, respectively. Left hand plots show on resonance depletion in black and off resonance natural loss in red, while right hand plots show the relative depletion of active species as a function of deposited energy. *Top row:* On resonance 590 cm^{-1}, off resonance 540 cm^{-1} (EI source). *Second row:* On resonance at 590 cm^{-1}, off resonance at 540 cm^{-1} (SIS Source). *Third row:* On resonance depletion at 1692 cm^{-1}, off resonance at 1623 cm^{-1}. *Bottom row:* On resonance at 3171 cm^{-1}, off resonance 3020 cm^{-1}. iv) Relative depletion at 3171 cm^{-1}. The errors shown are those arising from the fitting, with a further 5% uncertainty included for values presented in the main text, as outlined in Section 2.

Depletion saturation measurements were performed for a number of the observed bands, both to quantify the presence of isobaric impurities or excited electronic state of the $HCCS^+$ cation, and to ensure its consistency between the different bands and the different ion sources. Comparison of depletion measurements for EI and SIS sources has been performed for the $\sim$590 cm^{-1} feature, and additional depletion measurements were carried out, using the SIS source, in the intermediate and high wavenumber regions, at 1692 cm^{-1} and 3171 cm^{-1}. Results are shown in Figure S4. From the depletion results we estimate that 89(6)% of the intensity at the 1692 cm^{-1} and 3171 cm^{-1} bands is due to the triplet ground electronic state of the $HCCS^+$ cation, while for the feature at $\sim$590 cm^{-1}, the electronic state purity is given as 93(8)%, an average between the EI and SIS measurements.

Determination of the scaling factor

As reported in the main text, the computed harmonic line positions have been rescaled by a factor of 0.951. This has been independently determined by comparing the experimental spectrum with the calculated line positions from both the UB3LYP/cc-pVQZ level of theory (presented in the main text) and previously-reported calculations at the CCSD(T)/cc-pwCVQZ level[2]. From the NIST Computational Chemistry Comparison and Benchmark DataBase (CCCBDB), the reported scaling factor for the UB3LYP/cc-pVQZ is 0.9666[3], but no scaling factor is reported for the CCSD(T)/cc-pwCVQZ level.

Here, we have obtained a factor of 0.951 by comparing the observed, intense C-H stretching mode experimental band at 3171.4 cm^{-1} with the predicted line positions for this features obtained at both the UB3LYP level for the triplet (3333.3 cm^{-1}) and singlet states (3336.2 cm^{-1}) and the CCSD(T) level for the triplet (3339.5 cm^{-1}) and singlet (3340.8 cm^{-1}) states. Comparison of the UB3LYP spectrum with experiment using both the NIST scaling factor of 0.9666 and our scaling factor of 0.951 is shown in Figure S5.

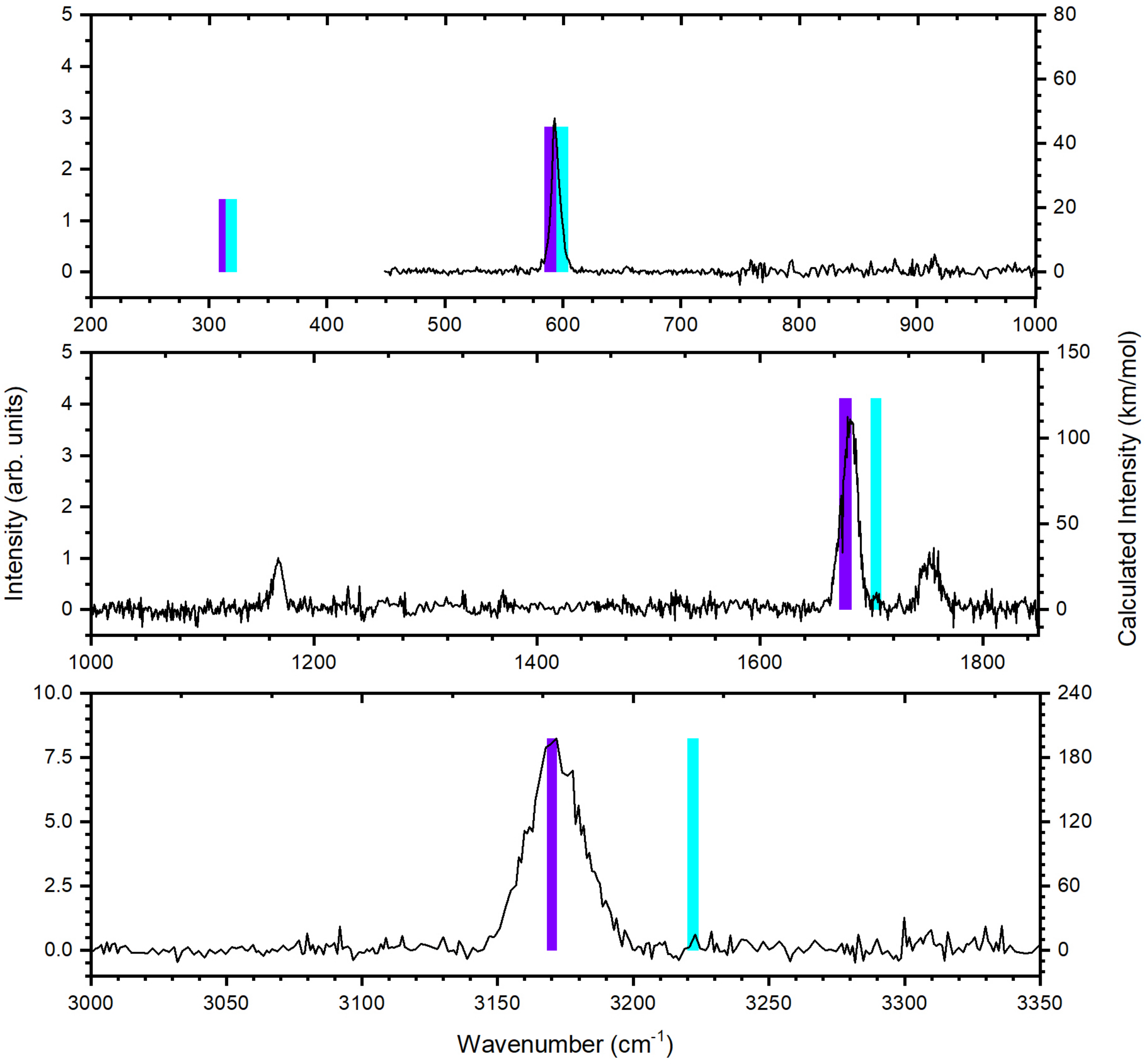

Figure S5: IR-PD vibrational spectrum of the H_2-tagged m/z 57 fragment from dissociative ionization of 2,5-dibromothiophene: experimental data is reported in black, while rescaled UB3LYP spectra of the bare ion using both the scaling factor of 0.951 determined in this work and that of 0.9666 from[3] are given by the purple and cyan lines, respectively.

Comparison of Calculated and Literature Ionization Energies

As mentioned in the main text, comparison of calculated ionization energies with experimental data to assess the performance of the UB3LYP/cc-pVQZ and G4 methods for several relevant sulphur-containing ions is provided in Table S1.

Table S1: Comparison of ionization energy values (in eV) for thiophene (c-CHCHCHCHS), 2,5-dibromothiophene (c-CBrCHCHCBrS), thioketene (H_2CCS), HCCS, methylsulphide (CH_3SH) and dimethylsulphide (CH_3SCH_3) calculated at the UB3LYP/cc-pVQZ and G4 levels of theory with reference values from NIST[4].

Species	NIST	UB3LYP/cc-pVQZ	G4
Thiophene	8.86 ± 0.02	8.67	8.95
2,5-Dibromothiophene	8.49[a]	8.19	8.46
Thioketene ($H_2C{=}C{=}S$)	8.77/8.89	8.84	8.89
$HC{=}C{=}S$	9.11[b]	9.08	9.23
Methylsulphide (CH_3SH)	9.439 ± 0.005	9.32	9.44
Dimethylsulphide (CH_3SCH_3)	8.69 ± 0.02	8.53	8.68

[a] Vertical ionization energy

[b] Theoretical value obtained at CCSD(T)/CBS+CV level of theory[2].

Comparison of calculated spectra from different levels of theory

As has also been already mentioned, the spectrum of the $HCCS^+$ ion (in both its triplet and singlet states) has already been modelled with the harmonic approximation at the ae-CCSDT(T)/cc-pwCVQZ level[2]. However, comparison of these calculations with our experimental results, while showing a good level of agreement in terms of predicted line positions, yielded very different relative intensities. For this reason, we have performed further harmonic calculations, both at the UB3LYP/cc-pVQZ level (harmonic and anharmonic) reported in the main text, and at the B2PLYP-D3BJ/aug-cc-pVTZ, CASPT2/cc-pVQZ CAS(6,4), fc-CCSD(T)/aug-cc-pVTZ, fc-CCSD(T)/cc-pV(T+d)Z and fc-MP2/aug-cc-pVTZ levels of theory. Comparison of the ae-CCSD(T), fc-CCSD(T) and fc-MP2 calculations with experiments is shown in Figure S6.

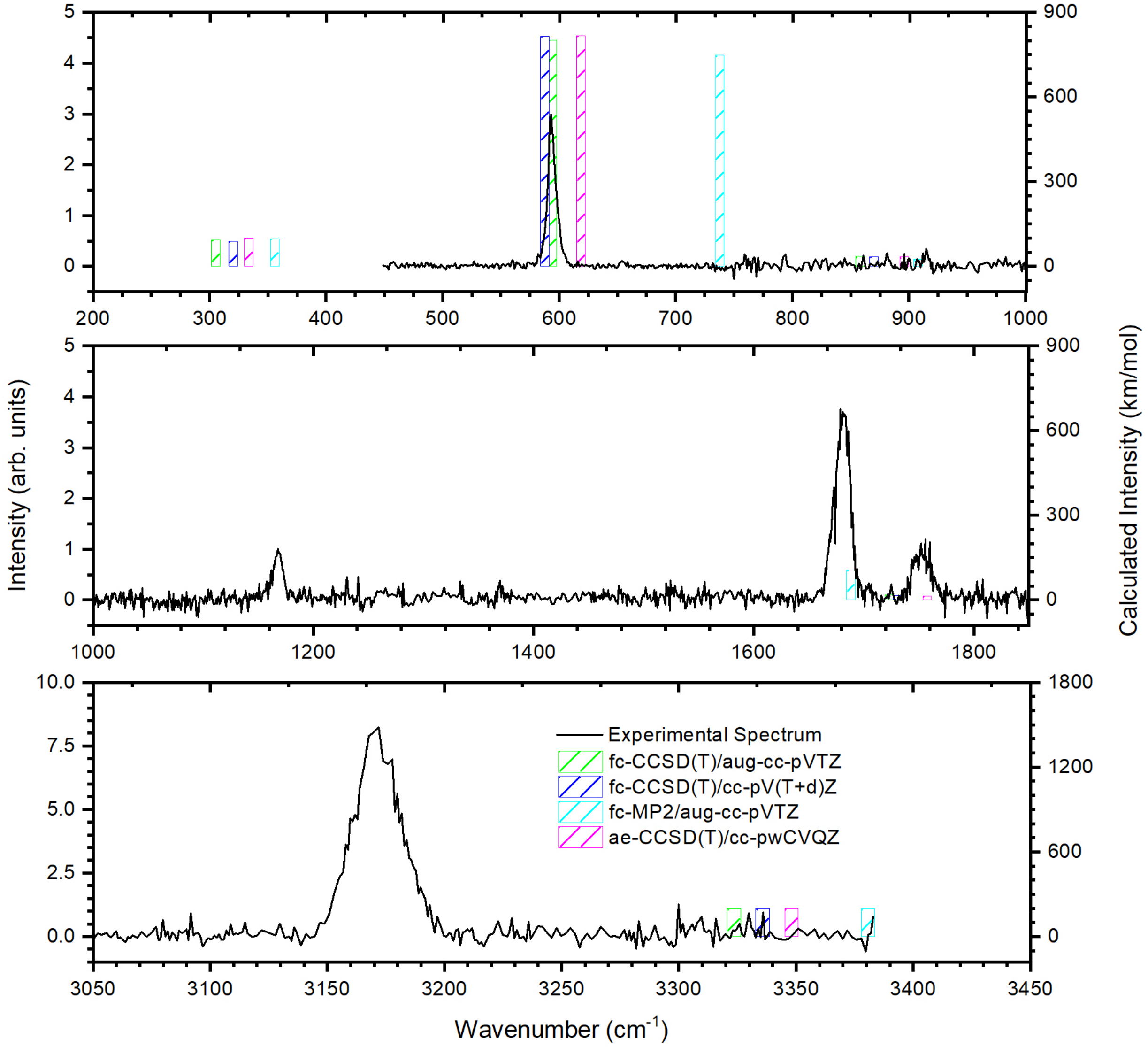

Figure S6: IR-PD vibrational spectrum of the H_2-tagged m/z 57 fragment from dissociative ionization of 2,5-dibromothiophene: experimental data are reported in black, while unscaled harmonic fc-CCSD(T)/aug-cc-pVTZ, fc-CCSD(T)/cc-pV(T+d)Z, fc-MP2/aug-cc-pVTZ and ae-CCSD(T)/cc-pwCVQZ calculated spectra of the bare ion are shown in green, purple, cyan and pink columns, respectively.

For all the fc-CCSD(T), fc-MP2 and ae-CCSD(T) spectra, we observe a significant over-prediction of the intensity of the H-C-C bending band at around 600 cm^{-1} relative to the other spectral features. The calculated frequencies of the bands are in reasonable agreement with experiment, with the exception of the position of the H-C-C bend predicted by the fc-MP2 level of theory. When we compare the experimental spectrum to the calculations

performed at the UB3LYP/cc-pVQZ, B2PLYP-D3BJ/aug-cc-pVTZ, and CASPT2/cc-pVQZ CAS(6,4) levels (see Figure S7), we note a much higher level of agreement, with the notable exception of the predicted frequencies and intensity of the C-C stretch at the CASPT2 level. In the main text, we have used the UB3LYP results in preference to the B2PLYP ones due to the slightly better match with the intensity of the C-C stretch, but both levels of theory accurately reproduce the experimental spectrum.

At the current stage, we are unable to identify the reason for the significant divergence between the spectral intensities predicted by different levels of theory, and significant further work is required to explore this. For instance, the H-C-C bending mode is very likely involved in Fermi and Darling-Dennison resonance with the first overtone of the C-C-S mode, which might be responsible for an intensity transfer contributing to the discrepancy noted above. While this issue deserved further investigations (that is anharmonic CCSD(T) calculations combined with generalized vibrational perturbation theory to second order, GVPT2), these are beyond the scope of the present study.

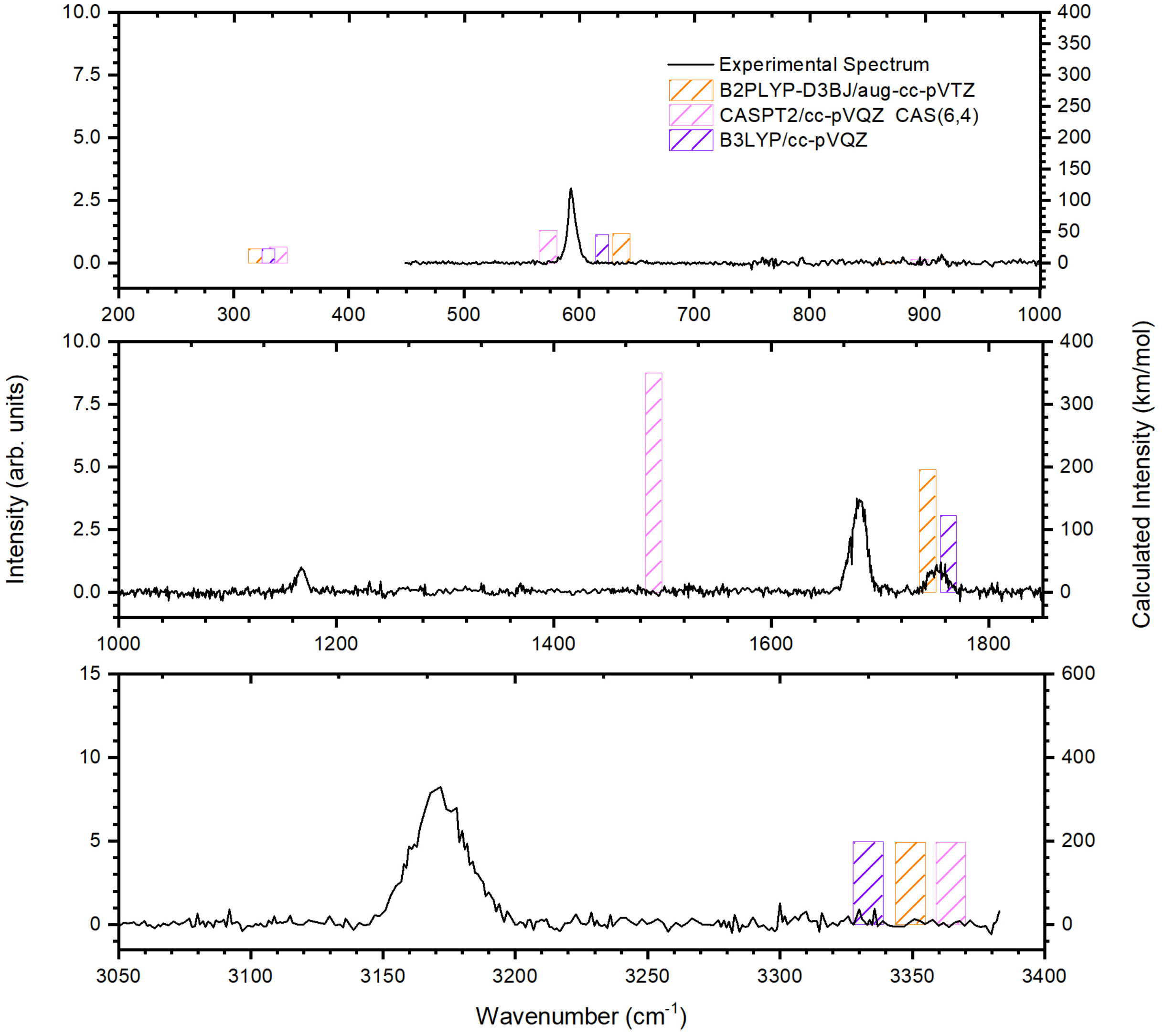

Figure S7: IR-PD vibrational spectrum of the H_2-tagged m/z 57 fragment from dissociative ionization of 2,5-dibromothiophene: experimental data are reported in black, while unscaled harmonic B2PLYP-D3BJ/aug-cc-pVTZ, CASPT2/cc-pVQZ CAS(6,4) and UB3LYP/cc-pVQZ calculated spectra of the bare ion are shown in orange, pink and purple columns, respectively.

Comparison of calculated spectra for tagged and bare $HCCS^+$

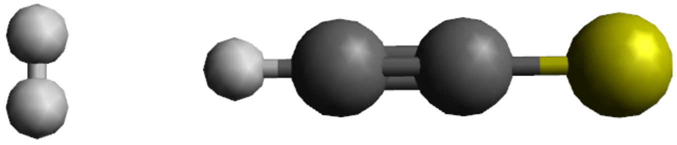

Figure S8: Geometry of the minimum energy structure of the H_2-$HCCS^+$ complex, calculated at the UB3LYP/cc-pVQZ level of theory.

In addition to calculations on the spectrum of the bare $HCCS^+$ ion, we have performed harmonic and anharmonic calculations on the geometries and spectra of the $HCCS^+$-H_2 complex. The lowest energy structure corresponds to a C_{2v} complex arising from a perpendicular approach towards the CH terminus of $HCCS^+$ (see Figure S8). Calculated binding energies for the lowest energy structure range from 5.7 to 7.9 kJ·mol^{-1} (equivalent to 477 to 660 cm^{-1}), depending on the basis set used. While the upper limit of this range extends beyond the observed H-C-C bending mode at 594 cm^{-1}, the observation of this mode is a strong indicator that the binding energy is below 7.1 kJ·mol^{-1} (594 cm^{-1}).

The calculated spectrum for the lowest-energy complex is shown alongside the equivalent UB3LYP calculations for the bare ion in Figure S9, while the calculated IR band positions and intensities are given in Table S2. The calculated scaled harmonic and anharmonic frequencies of the C-C stretch (1670/1711 cm^{-1}) are approximately unchanged from the calculated values for the bare ion (1675/1704 cm^{-1}). On the other hand, the calculated scaled harmonic and anharmonic frequencies of the C-H stretch (3095/3145 cm^{-1}) are shifted to lower wavenumbers compared to the bare ion (3170/3200 cm^{-1}). Both of these effects are as expected, since the influence of an H_2 at the CH terminus on the C-C stretching mode should be minimal, while the impact on the C-H stretch is naturally more significant. The ob-

served experimental frequency (3171 cm^{-1}) is in reasonable agreement with the anharmonic calculation for the tagged ion.

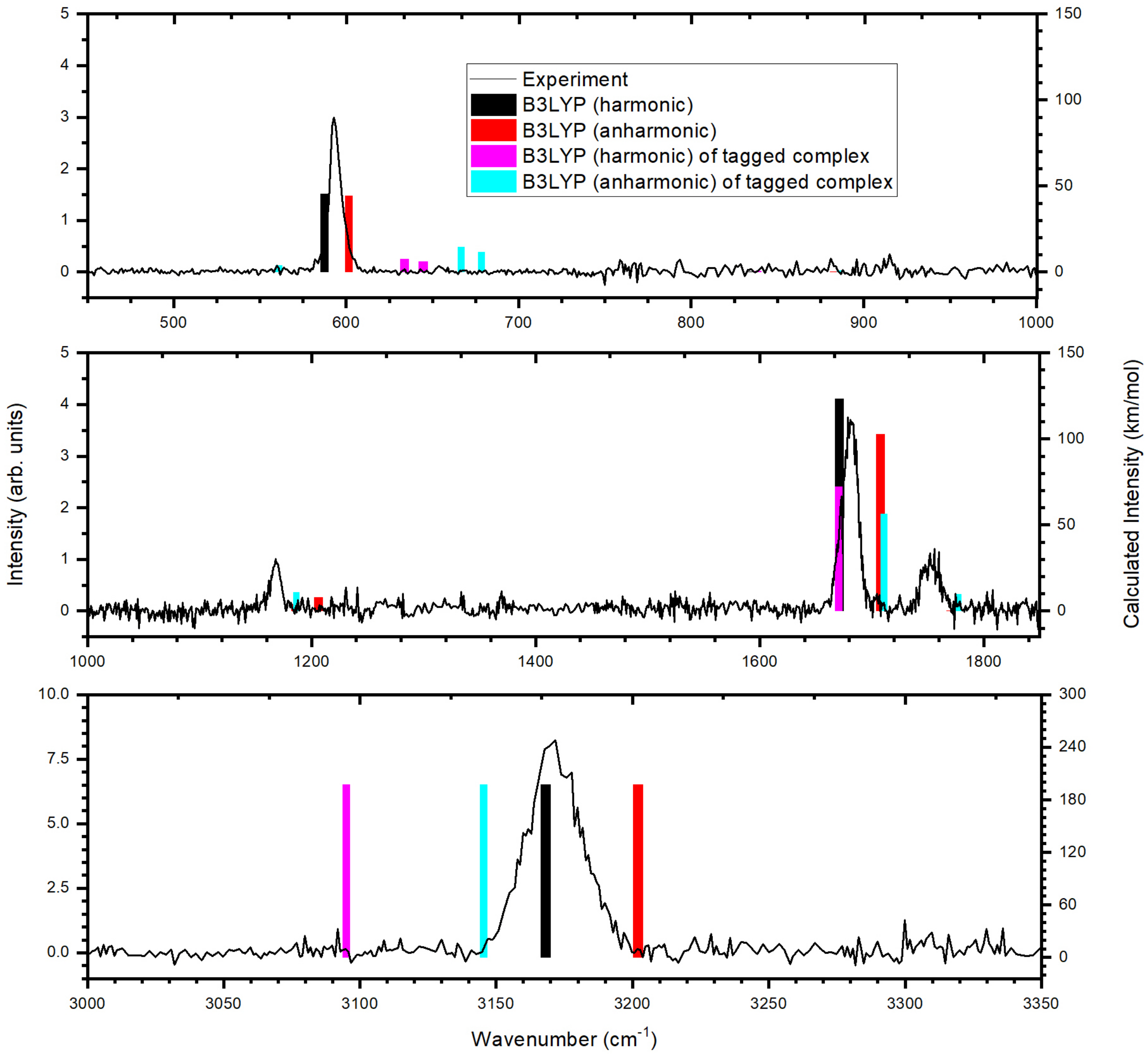

Figure S9: IR-PD vibrational spectrum of the H_2-tagged m/z 57 fragment from dissociative ionization of 2,5-dibromothiophene: experimental data is reported in black, while UB3LYP/cc-pVQZ calculations for both the bare $HCCS^+$ ion (harmonic in black and anharmonic in red) and the H_2-tagged complex (harmonic in pink and anharmonic in cyan) are shown as columns. Both harmonic spectra have been rescaled by a factor of 0.951.

The H-C-C bending mode in the lower wavenumber region is the mode for which the tagged and bare ion calculations differ the most. Due to the non-linear geometry of the complex, this mode is non-degenerate, leading to a splitting of the predicted features (ν_4

and ν_6 in Table S2) for both the harmonic and anharmonic calculations, in contrast to the single peak that is observed experimentally. Furthermore, this feature is notably shifted to higher wavenumbers with respect to both the experiment and the calculations for the bare ion.

It is also worth noting that for the overtone of the H-C-C bending mode, anharmonic calculations result in an out-of-plane mode (1186 cm^{-1}) that is even further shifted with respect to the in-plane mode (1452 cm^{-1}). However, the intensity of the in-plane overtone is several orders of magnitudes smaller than the out-of-plane overtone and so is not anticipated to be observed experimentally. The observed experimental band at 1168 cm^{-1} is therefore in reasonable agreement with the anharmonic tagged calculations of the H-C-C overtone(s).

As with the bare ion, the predicted intensity of the C-S stretch (ν_3) is very small, which explains the absence of an experimental feature in this region. However, the overtone of this band ($2\nu_3$) has a much higher predicted intensity (19.2 km/mol) than the fundamental mode (2.1 km/mol), with the predicted anharmonic position of this overtone (1777 cm^{-1}) being in good agreement with the observed experimental feature at 1753 cm^{-1}. Notably, the anharmonic calculations of the tagged complex effectively reproduce both the positions and relative intensities of the two observed overtones. It is unclear whether the observed features are enhanced by the tagging, but the overall better agreement between the experimental spectrum and the predicted spectra of the bare ion indicates the overall contribution from the tag is minimal.

Finally, we note that while the harmonic calculations for both the tagged complex and the bare ion predict the C-C-S bending mode to lie outside the wavenumber range that we have explored experimentally, the anharmonic calculations of the tagged complex predict a significant shift to higher wavenumbers (560.7 cm^{-1}), albeit with a comparatively small intensity (7.3 km/mol). However, no clear feature consistent with this prediction is observed experimentally.

Table S2: Calculated IR band positions (in cm^{-1}) and intensities (in km mol^{-1}) for the triplet ($^3\Sigma^-$) C_{2v} complex of H_2 - $HCCS^+$.

Mode	Symmetry	Calc. Pos.[b] cm^{-1}	Calc. Int.[b] km·mol^{-1}	Calc. Pos.[c] cm^{-1}	Calc. Int.[c] km/mol
C-C-S bend in-plane (ν_7)	B_2	317	9.8	560.7	7.3
C-C-S bend out-of-plane (ν_5)	B_1	319	8.2	390.4	12.4
H-C-C bend out-of-plane (ν_4)	B_1	634	16.8	666	28.3
H-C-C bend in-plane (ν_6)	B_2	644	14.1	678	22.3
C-S stretch (ν_3)	A_1	838	1.4	886	2.1
H-C-C bend out-of-plane, overtone ($2\nu_4$)	B_1	1268	-	1186	21.2
H-C-C bend in-plane, overtone ($2\nu_6$)	B_1	1288	-	1452	0.004
C-C stretch (ν_2)	A_1	1670	164.7	1711	110.0
C-S stretch, overtone ($2\nu_3$)	A_1	1676	-	1777	19.2
C-H stretch (ν_1)	A_1	3095	450.5	3145	385.8

[a] Overtone positions obtained form the harmonic calculations are multiples of the fundamental position.

[b] This work, UB3LYP/cc-pVQZ (harmonic). For comparison with experimental results, a vibrational scaling factor of 0.951 has been applied to the calculated frequencies.

[c] This work, UB3LYP/cc-pVQZ (anharmonic).

Calculations for the Singlet Excited State

Frequency calculations for the singlet $HCCS^+$ cation at the B3LYP/cc-pVQZ level of theory using the Gaussian 16 default settings led to an erroneous set of vibrational frequencies (four different bending modes instead of the expected two pairs of degenerate modes). To obtain more accurate vibrational frequencies for this linear, $C_{\infty v}$ symmetric species, the initial guess for the Hartree-Fock wavefunction was made using the INDO method for hydrogen and the Hückel method for carbon and sulfur, with the HOMO and LUMO orbitals mixed to destroy both α-β and spatial symmetries. The stable wave function that was thus obtained was then used for subsequent geometry optimization and harmonic and anharmonic frequency calculations. The analogous procedure was also used in the case of G4 calculations to obtain higher accuracy energies for this species. For the overtone of the H-C-C bend, the anharmonic wavenumber of the $l = \pm 2$ mode is the average of the predicted anharmonic wavenumbers of the $l = 2$ and $l = -2$ modes.

Structure diagrams and atom coordinates

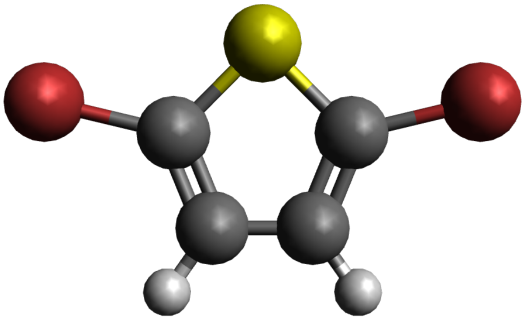

Figure S10: Structure diagram for the 2,5-dibromothiophene cation optimized at the G4 level of theory

Table S3: Atom coordinates for the 2,5-dibromothiophene cation in angstroms (Å) optimized at the G4 level of theory

Atom Type	X Coordinate (Å)	Y Coordinate (Å)	Z Coordinate (Å)
C_1	0.0000000000	1.2250480000	0.5466241111
C_2	0.0000000000	0.6904500000	-0.7611638889
C_3	0.0000000000	-0.6904500000	-0.7611638889
C_4	0.0000000000	-1.2250480000	0.5466241111
S_1	0.0000000000	0.0000000000	1.7785311111
H_1	0.0000000000	1.3145580000	-1.6457808889
H_2	0.0000000000	-1.3145580000	-1.6457808889
Br_1	0.0000000000	3.0024500000	0.9710551111
Br_2	0.0000000000	-3.0024500000	0.9710551111

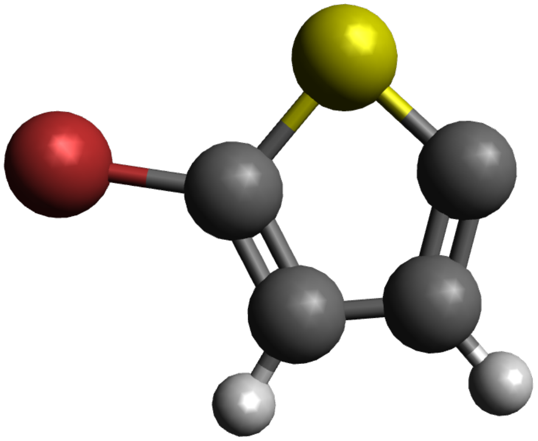

Figure S11: Structure diagram for the intermediate structure A_t optimized at the G4 level of theory

Table S4: Atom coordinates for the intermediate structure A_t in angstroms (Å) optimized at the G4 level of theory

Atom Type	X Coordinate (Å)	Y Coordinate (Å)	Z Coordinate (Å)
C_1	1.6451656250	0.3366273750	0.0000000000
C_2	0.5054156250	1.1495563750	0.0000000000
C_3	-0.6266453750	0.3492143750	0.0000000000
C_4	-0.3109253750	-1.0364406250	0.0000000000
S_1	1.4293736250	-1.3406426250	0.0000000000
H_1	0.5191816250	2.2321643750	0.0000000000
H_2	-1.6484213750	0.7103643750	0.0000000000
Br_1	-1.5131443750	-2.4008436250	0.0000000000

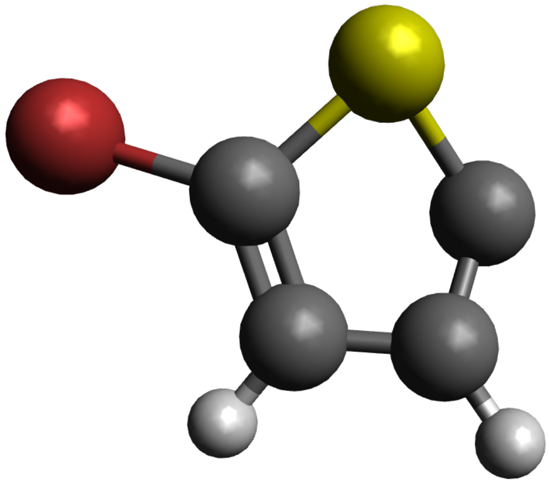

Figure S12: Structure diagram for the intermediate structure A_s optimized at the G4 level of theory

Table S5: Atom coordinates for the intermediate structure A_s in angstroms (Å) optimized at the G4 level of theory

Atom Type	X Coordinate (Å)	Y Coordinate (Å)	Z Coordinate (Å)
C_1	1.2478756250	-0.8524695000	0.4133756250
C_2	1.1998546250	0.4480975000	-0.0212843750
C_3	-0.1811323750	0.6856625000	-0.0960903750
C_4	-0.9307803750	-0.5163025000	-0.1818913750
S_1	0.0551396250	-1.9695455000	-0.0771223750
H_1	2.0083696250	1.1633885000	-0.0961233750
H_2	-0.6573083750	1.6555235000	0.0050236250
Br_1	-2.7420183750	-0.6143545000	0.0541126250

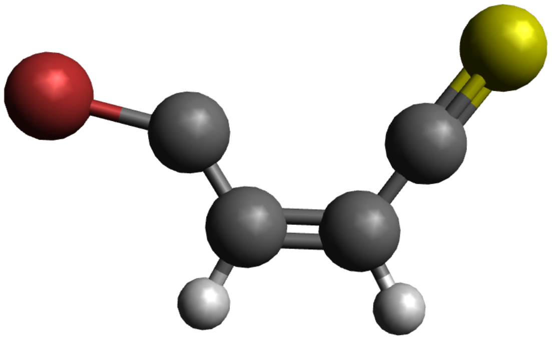

Figure S13: Structure diagram for the intermediate structure B_t optimized at the G4 level of theory

Table S6: Atom coordinates for the intermediate structure B_t in angstroms (Å) optimized at the G4 level of theory

Atom Type	X Coordinate (Å)	Y Coordinate (Å)	Z Coordinate (Å)
C_1	-0.0102805000	-1.6832385000	0.0000000000
C_2	0.7644035000	-0.5667155000	0.0000000000
C_3	0.2796575000	0.7554555000	0.0000000000
C_4	-1.0511775000	1.0656215000	0.0000000000
S_1	-0.8359345000	-2.9551955000	0.0000000000
H_1	1.8408675000	-0.7378085000	0.0000000000
H_2	1.0217185000	1.5549245000	0.0000000000
Br_1	-2.0092545000	2.5669565000	0.0000000000

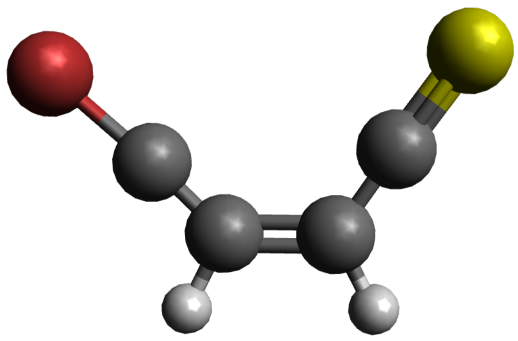

Figure S14: Structure diagram for the intermediate structure B_s optimized at the G4 level of theory

Table S7: Atom coordinates for the intermediate structure B_s in angstroms (Å) optimized at the G4 level of theory

Atom Type	X Coordinate (Å)	Y Coordinate (Å)	Z Coordinate (Å)
C_1	0.5580358750	-1.5629255000	0.0000000000
C_2	0.9380338750	-0.2835595000	0.0000000000
C_3	0.0397698750	0.8331635000	0.0000000000
C_4	-1.2516011250	0.9854855000	0.0000000000
S_1	0.1593878750	-3.0404335000	0.0000000000
H_1	2.0008318750	-0.0650585000	0.0000000000
H_2	0.5021678750	1.8354295000	0.0000000000
Br_1	-2.9466261250	1.2978985000	0.0000000000

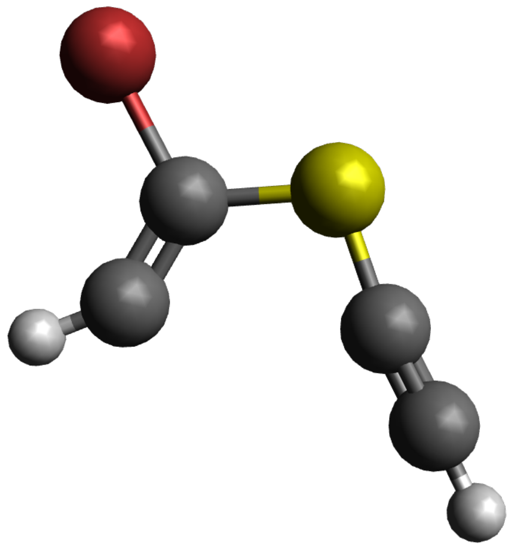

Figure S15: Structure diagram for the intermediate structure C_t optimized at the G4 level of theory

Table S8: Atom coordinates for the intermediate structure C_t in angstroms (Å) optimized at the G4 level of theory

Atom Type	X Coordinate (Å)	Y Coordinate (Å)	Z Coordinate (Å)
C_1	1.2723673750	-0.7324417500	0.0000000000
C_2	2.4284783750	-0.3608397500	0.0000000000
C_3	-0.9955376250	1.1683102500	0.0000000000
C_4	-1.3501096250	-0.1180657500	0.0000000000
S_1	-0.2204926250	-1.4428677500	0.0000000000
H_1	3.4422843750	-0.0197297500	0.0000000000
H_2	-1.4173716250	2.1638052500	0.0000000000
Br_1	-3.1596186250	-0.6581707500	0.0000000000

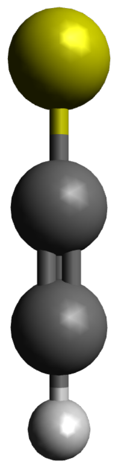

Figure S16: Structure diagram for the triplet $HCCS^+$ product ion optimized at the G4 level of theory

Table S9: Atom coordinates for the triplet $HCCS^+$ product ion in angstroms (Å) optimized at the G4 level of theory

Atom Type	X Coordinate (Å)	Y Coordinate (Å)	Z Coordinate (Å)
C_1	0.0000000000	0.0000000000	-0.7514710000
C_2	0.0000000000	0.0000000000	0.5075290000
H_1	0.0000000000	0.0000000000	-1.8275180000
S_1	0.0000000000	0.0000000000	2.0714600000

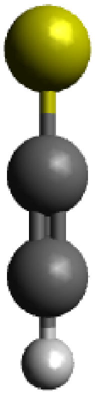

Figure S17: Structure diagram for the singlet $HCCS^+$ product ion optimized at the G4 level of theory

Table S10: Atom coordinates for the singlet $HCCS^+$ product ion in angstroms (Å) optimized at the G4 level of theory

Atom Type	X Coordinate (Å)	Y Coordinate (Å)	Z Coordinate (Å)
C_1	0.0000000000	0.0000000000	-0.7507817500
C_2	0.0000000000	0.0000000000	0.5058592500
H_1	0.0000000000	0.0000000000	-1.8267107500
S_1	0.0000000000	0.0000000000	2.0716332500

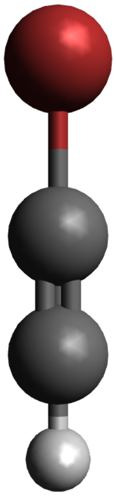

Figure S18: Structure diagram for the HCCSBr product neutral optimized at the G4 level of theory

Table S11: Atom coordinates for the HCCSBr product neutral in angstroms (Å) optimized at the G4 level of theory

Atom Type	X Coordinate (Å)	Y Coordinate (Å)	Z Coordinate (Å)
Br_1	0.0000000000	0.0000000000	2.2102027500
C_1	0.0000000000	0.0000000000	0.4185077500
C_2	0.0000000000	0.0000000000	-0.7833932500
H_1	0.0000000000	0.0000000000	-1.8453172500

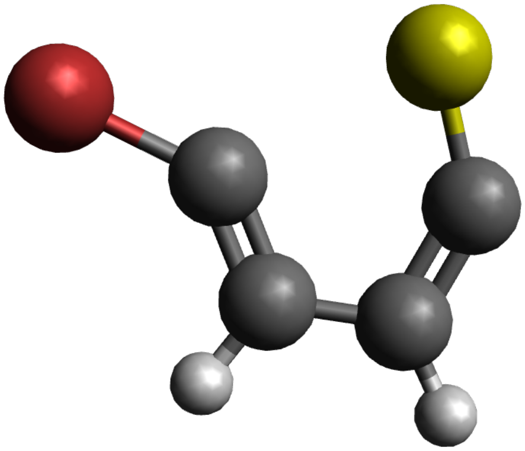

Figure S19: Structure diagram for the A_t-B_t transition state optimized at the G4 level of theory

Table S12: Atom coordinates for the A_t-B_t transition state in angstroms (Å) optimized at the G4 level of theory

Atom Type	X Coordinate (Å)	Y Coordinate (Å)	Z Coordinate (Å)
C_1	1.4955663750	-0.7301488750	0.0000897500
C_2	1.0638093750	0.5806401250	0.0000347500
C_3	-0.3499196250	0.6509771250	-0.0000352500
C_4	-1.1003296250	-0.4936858750	-0.0000402500
S_1	0.8499743750	-2.1346678750	0.0001037500
H_1	1.7034163750	1.4536691250	0.0000477500
H_2	-0.8432996250	1.6214001250	-0.0000922500
Br_1	-2.8192176250	-0.9481838750	-0.0001082500

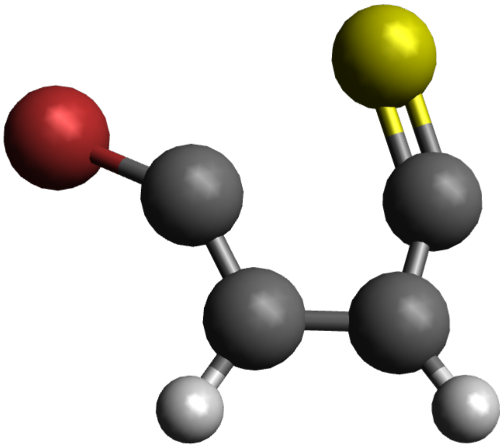

Figure S20: Structure diagram for the A_s-B_s transition state optimized at the G4 level of theory

Table S13: Atom coordinates for the A_s-B_s transition state in angstroms (Å) optimized at the G4 level of theory

Atom Type	X Coordinate (Å)	Y Coordinate (Å)	Z Coordinate (Å)
C_1	-1.2350495000	-0.8222635000	-0.3027256250
C_2	-1.1167865000	0.5088035000	-0.1312356250
C_3	0.2733035000	0.7272335000	0.0666423750
C_4	1.0704965000	-0.3487845000	0.3630273750
S_1	-0.5320685000	-2.1698645000	0.0427603750
H_1	-1.8992645000	1.2530515000	-0.0869666250
H_2	0.7191105000	1.7204515000	0.1286293750
Br_1	2.7202585000	-0.8686275000	-0.0801316250

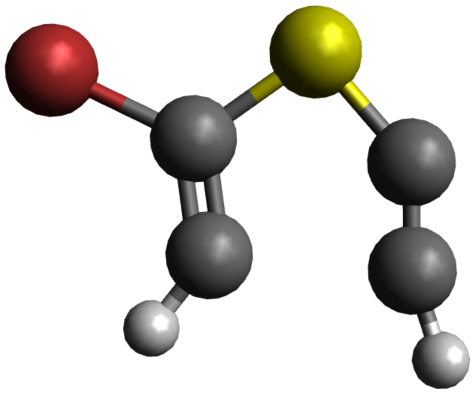

Figure S21: Structure diagram for the A_t-C_t transition state optimized at the G4 level of theory

Table S14: Atom coordinates for the A_t-C_t transition state in angstroms (Å) optimized at the G4 level of theory

Atom Type	X Coordinate (Å)	Y Coordinate (Å)	Z Coordinate (Å)
C_1	1.3473308750	-0.9877157500	-0.0000440000
C_2	1.7654508750	0.1755622500	-0.0000010000
C_3	-0.4906011250	0.8018642500	0.0000330000
C_4	-1.1165321250	-0.4089997500	-0.0000110000
S_1	-0.1129511250	-1.8281607500	-0.0000700000
H_1	2.4104238750	1.0317252500	0.0000300000
H_2	-0.8405391250	1.8286192500	0.0000740000
Br_1	-2.9625821250	-0.6128947500	-0.0000110000

References

(1) Diprose, J. A.; Steenbakkers, K.; Michielan, M.; Polášek, M.; Ascenzi, D.; Brünken, S.; Romanzin, C.; Heazlewood, B. R.; Richardson, V. Selective formation and spectroscopic characterization of the $H_2CCS^{\bullet+}$ radical cation via dissociative ionization of thiophene. *J. Chem. Phys.* **2025**, *162*, 164304.

(2) Puzzarini, C. A theoretical investigation on the HCCS radical and its ions. *Chem. Phys.* **2008**, *346*, 45–52.

(3) NIST Computational Chemistry Comparison and Benchmark Database. NIST Standard Reference Database Number 101 Editor: Russell D. Johnson III `http://cccbdb.nist.gov/`, 2022; Release 22, May.

(4) Linstrom, P. J.; Mallard, W. G. NIST Chemistry WebBook - Standard Reference Database n. 69. [Online], accessed April 2024; `http://webbook.nist.gov`.
